# Assessing the Feasibility and Effectiveness of a Linkage Into Mental Health Care Program for Adults Affected by Hurricane Sandy

**DOI:** 10.1017/dmp.2022.176

**Published:** 2022-09-05

**Authors:** Rebecca M Schwartz, Samantha S Corley, Rehana Rasul, Kristin G Bevilacqua, Adam Gonzalez, Christina Gillezeau, Wil Lieberman-Cribbin, Emanuela Taioli

**Affiliations:** 1 Department of Occupational Medicine, Epidemiology and Prevention, Northwell Health, Great Neck, NY, USA; 2 Feinstein Institutes for Medical Research, Manhasset, NY, USA; 3 Zucker School of Medicine at Hofstra/Northwell, Hempstead, NY, USA; 4 Institute for Translational Epidemiology and Department of Population Health Science and Policy, Icahn School of Medicine at Mount Sinai, New York, NY, USA; 5 Center for Traumatic Stress, Resilience and Recovery at Northwell Health, New Hyde Park, NY; 6 Department of Psychiatry & Behavioral Health at Stony Brook University School of Medicine, Stony Brook University, Stony Brook, NY, USA

**Keywords:** hurricanes, mental health care, anxiety

## Abstract

**Objective:**

Research indicates that greater exposure to Hurricane Sandy is associated with increased mental health difficulties. This study examined whether Project Restoration, a program that linked adults into mental health care (L2C), was effective in reducing post-Sandy mental health difficulties as compared to a cohort of adults matched on mental health difficulties that were not linked into post-Sandy mental health care.

**Methods:**

Project Restoration participants (n = 52) with elevated self-reported mental health difficulties had the option to enroll into L2C. Project LIGHT (n = 63) used similar methodologies but did not have a L2C component and served as the matched control group.

**Results:**

Multivariable modeling showed significant decreases in all mental health difficulties except for depression in the Project Restoration group, whereas there were no significant decreases in LIGHT. The decrease in anxiety from baseline to follow-up was significantly greater for Project Restoration as compared to LIGHT.

**Conclusion:**

Findings confirm the powerful impact community outreach and treatment have on reducing mental health difficulties after a disaster.

## Introduction

Hurricane Sandy made landfall in New York on October 29, 2012 as the largest Atlantic storm in recorded history.^1^ Coastlines along New York City (NYC), Long Island (LI), and the Hudson River Valley (HRV) experienced storm surges of 2 to 9 feet above ground level with a maximum sustained wind of 75 mph in eastern LI.^2^ As a result of widespread flooding and power outages, an estimated 370,000 people were evacuated and over 300,000 homes were damaged in New York State.^2^ Of the 117 hurricane-related deaths recorded by the American Red Cross, 53 occurred in New York.^3^ On October 30, a Major Disaster Declaration for New York was made by the Federal Emergency Management Agency and disaster response centers opened in the greater NYC area on November 1, 2020.^4^ However, many residents continued without power for days or weeks, while others did not return to their homes for months after the storm.^2^ This held especially true for the neighborhoods in the Rockaways section of NYC, a location highly affected by Hurricane Sandy where high flood waters inundated many low-lying areas. This flooding caused widespread damage and limited the ability of first responders to access a large fire in Breezy Point, an area of the Rockaways. As a result of this, the fire destroyed over 100 homes before it was contained.^2^ In addition to the cost of property damage, flooding and natural disasters can have irreversible effects on ecological systems, physical health, and human life. These impacts of climate change are likely to continue to put low-lying areas, such as the Rockaways, at increased risk for significant deleterious health effects at the population level.^5^

The impacts of natural disasters such as the Hurricane Sandy on mental health are well-documented.^6–13^ Exposure to a natural disaster has been shown to increase the risk of developing symptoms of anxiety, depression, and/or post-traumatic stress disorder (PTSD) immediately after the event and later in life,^10,13–16^ increase suicidal ideation and attempts, and increase substance use.^14,17^ The psychological impacts of natural disasters are strongly associated with the type and intensity of exposure experienced by an individual,^14,18,19^ with certain subpopulations including those of low-income, black race, and female gender, at a greater risk for mental health difficulties.^20–22^ After its landfall in the northeastern parts of the United States, Hurricane Sandy contributed to increased symptoms of PTSD and depression among survivors, and had long-lasting mental health effects, still detectable up to 4 years after the storm.^6,10,13,23,24^

The abrupt nature of disasters and the need to prioritize physical safety and well-being in the wake of a disaster have limited the implementation and evaluation of disaster mental health interventions.^25,26^ Interventions, particularly those addressing mental health, tend to be short-term in nature and limited to psychological first aid, psychological debriefing or crisis counseling; few interventions have linked adult survivors to long-term, formal mental health treatment.^26–29^ Due in part to these constraints, North and Pfefferbaum’s systematic review of mental health responses to community disasters found disaster emergency and medical response literature lacking in evidence and methodological rigor.^26^ This is the first study to examine the effects of linkage to a mental health care program for individuals impacted by Hurricane Sandy. We compare findings to a group of individuals who also display mental health symptoms and were impacted by Hurricane Sandy but were not linked to care after the storm. This effectiveness-study aims to test the hypothesis that, for individuals reporting symptoms of mental health difficulties, engaging in a mental health treatment in the wake of a natural disaster is associated with decreased mental health symptoms including perceived stress, depression, anxiety, and PTSD, following treatment as compared to a sample of participants who were not offered linkage to mental health care.

## Methods

### Participants and procedures

The present study uses baseline and follow-up data from a series of projects that were new and externally funded after Hurricane Sandy. Leaders in Gathering Hope Together (LIGHT) was a federally funded grant that collected baseline mental health impact survey data to examine the impact of Hurricane Sandy on symptoms of mental health difficulties. LIGHT gathered follow-up data from their research participants using an additional federally funded grant specifically aimed at longitudinally following the original LIGHT cohort. LIGHT recruited participants from Nassau County, Suffolk County, and boroughs of NYC including Queens and Staten Island from October 23, 2013, to February 25, 2015. Project Restoration (PR) was a third grant that was foundationally funded which also aimed to examine the impact of Hurricane Sandy on symptoms of mental health difficulties. The PR grant funded survey data collection for both baseline and follow-up time points for a separate cohort of participants (who did not participate in LIGHT) and who resided in the Rockaways during Hurricane Sandy. Unlike LIGHT, PR included a Linkage to Care (L2C) program for participants who screened elevated on the mental health screening measures during baseline data collection. PR was conducted from June 5, 2014, to August 9, 2016 and focused exclusively on residents of the Rockaways, Queens (NYC). Both LIGHT and PR utilized similar recruitment methodology and identical survey instruments to be able to compare data across their cohorts.

In both LIGHT and PR, convenience sampling methods were used to recruit participants at a variety of locations such as street fairs, libraries, and supermarkets. Research staff worked in partnership with local community agencies and governmental groups to coordinate recruitment events, including distributing flyers with the date, time, and location of the event for both LIGHT and PR. Eligibility criteria for LIGHT and PR were similar as participants were eligible for LIGHT if they spoke English or Spanish, did not have cognitive impairments that prevented them from providing informed consent, were at least 18 years old, and lived in LI/NYC during Hurricane Sandy. PR had the same eligibility requirements but participants had to reside in the Rockaways at the time of Hurricane Sandy. Eligible participants for both LIGHT and PR were compensated for their participation at each time point. The studies were approved by IRB #13–499B at Northwell Health and #15–00513 at Mount Sinai. This present study utilizes baseline and follow-up data from both PR (which included the L2C program) and LIGHT (which did not include the L2C program) to assess the effectiveness of the L2C program on symptoms of mental health difficulties.

### Linkage to Care (L2C) intervention

The L2C program entailed: (1) establishing contact with the participant, (2) gathering information to assess barriers to care and determine linkage to mental health care tailored to the participant, (3) attending a mental health care appointment, and (4) following-up with participant to ensure that barriers to engagement in care were addressed.

#### Establishing contact

After recruitment, the coordinator called each consenting participant to brief them on L2C, and then obtained medical release forms. Potential L2C participants were contacted up to 5 times at each stage by the study coordinator. If they indicated interest in L2C, they were in the L2C portion of outreach, and the coordinator attempted to link them into care. If at any point in the outreach process, a participant indicated they were still interested, up to 5 more attempts were made to contact the participant.

#### Identifying and addressing barriers

Specific mental health care needs and barriers to care were identified to link participants to mental health care. The coordinator either addressed the barrier or connected the participant with an organization that would be able to address it. Coordinators either linked participants who were not already in care with providers in their community or addressed any barriers to provision of care for participants who were already enrolled in treatment. If the barriers were financial (such as cost of transportation to the mental health care provider), the coordinator reimbursed the participant for their expenses.

#### Linkage to care/attending an appointment

The coordinator contacted participants by phone to link them to a mental health care provider within the community and assisted them in attending at least 1 mental health treatment appointment. The coordinator received signed permission from participants to communicate with the potential mental health provider, allowing the coordinator to advocate on behalf of participants. Once the participant went to at least 1 appointment, they were considered successfully linked. Participants were linked with providers in the Rockaways and the larger NY metropolitan area. These providers included state-funded post-Sandy mental health providers, clinical psychologists in private practice, substance abuse clinics, and mental health clinics offering a range of therapy options. The coordinator followed up with participants once they were linked to care to ensure that barriers to care could continue to be addressed. This facilitated the continuation of treatment session attendance. Study staff contacted L2C participants 6 months after the date they linked into care to administer a follow-up questionnaire. More detailed information on the L2C intervention has been published elsewhere.^30^

### Treatment group inclusion criteria

In PR, eligible participants were given the option to enroll in a program that linked them with a local mental health care provider (L2C). Eligibility was determined through the presence of elevated symptom scores on the baseline questionnaire administered onsite using mental health and substance abuse screening criteria (S1 Table). PR participants who were linked into care were asked to complete the questionnaire again at a follow-up period approximately 6 months later. Therefore, the treatment group for the current analysis consists of those who received L2C and completed the follow-up questionnaire (n = 52).

### LIGHT control group inclusion criteria

Participants in LIGHT were not offered the L2C program. The LIGHT study did not require participants to have increased levels of symptoms of mental health difficulties, however, these participants completed the same mental health and substance abuse questionnaires as the participants in the PR study. Only those participants with elevated symptom scores were included in the current analyses, to ensure that the LIGHT control group had similar baseline mental health to the PR group (see S1 Table for inclusion rubric). Participants in LIGHT were also asked to complete questionnaires at a follow-up period. Therefore, the control group for the current analysis consists of LIGHT participants who have elevated mental health symptoms (as per the rubric used for L2C) and who also completed a follow-up questionnaire (n = 63). Please see S2 Table for differences between participants whose data were included in the current analyses and those whose data were not.

### Questionnaire

On baseline and follow-up questionnaires, participants answered questions about demographics, history of mental health difficulties, current mental health treatment status, hurricane exposure, and months elapsed since Hurricane Sandy at the time of survey completion. The questionnaire also included validated, psychometrically sound instruments to measure symptoms of mental health difficulties, including symptoms of PTSD, anxiety, depression, and perceived stress (see S1 Table).

### Outcomes

Outcomes studied included symptoms of anxiety, depression, perceived stress, and PTSD. Anxiety and depression symptoms were measured using the Patient Health Questionnaire-4 (PHQ-4),^31^ which has been used in previous disaster studies^10,13,32,33^ and provides a summed score of symptoms (range: 0 – 6). Perceived stress was measured using the Perceived Stress Scale (PSS-10), a 10-item scale used to assess current stress.^34^ PTSD symptom scores were assessed by summing all items (range: 17 – 85) from the self-reported Post-traumatic Stress Disorder Checklist-Specific (PCL-S).^35^ The measure contains 17 questions about PTSD symptoms (based on the DSM-IV) in reference to a specific traumatic occurrence, in this case, Hurricane Sandy.

In addition to these, current smoking status, drug use, and problem alcohol use were assessed. Current smoking status (Yes/No) was defined as currently smoking daily. Drug use (Yes/No) was defined as any use of a recreational drug or prescription medication for non-medical reasons in the past year.^36^ Problem alcohol use (Yes/No) was defined as the indication of heavy drinking (at least 8 or 15 drinks per week, for females and males respectively), or the indication of binge drinking (having at least 4 or 5 drinks in a day in the past year, for females and males, respectively).^37^

Outcomes were measured from surveys administered at 2 periods: baseline and follow-up. Baseline measures were taken before start of the L2C intervention for PR participants, or at time of study enrollment for LIGHT participants. Follow-up surveys were administered to the L2C group approximately 6 months after linking into care and approximately 17 months after baseline measures for the LIGHT control group.

### Additional study variables

Demographics [age, gender (Male/Female), race (White/Other), ethnicity (Hispanic/Non-Hispanic), education level (< high school (HS)/≥ HS)], prior mental health history (Yes No), current mental health treatment status (Yes/No), hurricane exposure score, the difference in time (months) between Hurricane Sandy and baseline survey completion, and the difference in time (months) between baseline and follow-up surveys were also studied. The hurricane exposure score was calculated as the sum of 30 potential exposures due to Hurricane Sandy, such as loss of property, loss of working hours, injury, displacement, and loss of pets or family.^10,11^

### Analysis strategy

Baseline characteristics were described for both the L2C and LIGHT control groups. Frequency and percent were reported for categorical variables while mean and standard deviation (SD) or median (Med) and interquartile range (IQR) were provided for continuous variables. Group differences were assessed using Chi-square tests for categorical variables and Mann-Whitney Rank Sum tests for continuous variables. Differences in outcomes between baseline and follow-up were also assessed using McNemar’s test or Wilcoxon sign rank test for categorical and continuous measures, respectively, for each group.

Linear mixed models, accounting for repeated measures, were used to determine if the association between period (baseline and follow-up) and mental health outcomes differed between L2C and control groups. An interaction between L2C group and time was included to determine if there was a decrease in outcomes from baseline to follow up within each group. All models were adjusted for age, gender, race, ethnicity, education level, prior mental health history, hurricane exposure score, difference in time (months) between Hurricane Sandy and baseline survey completion, and difference in time (months) between baseline and follow-up surveys. As current mental health treatment status was missing for 10 LIGHT participants, it was not included in the main models. A sensitivity analysis including mental health treatment did not return significantly different results.

Adjusted parameter estimates (B) and 95% confidence intervals (CI) were reported for linear regression models. A Benjamini-Hochberg correction was applied to all confidence intervals and *P* values from testing subgroups using the interaction term to account for multiple hypothesis testing. Significance was reported as *P*< 0.05. All analyses were conducted using SAS 9.4 (SAS Institute Inc., Cary, North Carolina, USA).^38^

## Results

Table 1 presents descriptive information for the 15 participants included in the study (LIGHT control n = 63; L2C n = 52) and indicates where there are baseline differences between the 2 groups. Of note, the L2C group had a higher proportion of participants with a history of mental health difficulties (65.38% vs. 28.57%, *P*< 0.001) and a higher proportion of participants currently in treatment (67.31% vs. 15.09%, *P*= 0.001). Although the same inclusion rubric was used for both groups, for all primary mental health outcomes, mean symptom scores were higher in the L2C group compared to the control group at baseline (Table 2). Proportions of substance abuse at baseline were significantly higher for the L2C group compared to the control group, except for problem alcohol use.

The unadjusted paired analysis indicated that there were significant decreases in all mental health outcomes from baseline to follow-up in the L2C group, whereas the LIGHT control group only evidenced significant decreases from baseline to follow-up in PSS (stress) and PTSD symptom scores (Table 2).

### Multivariable results

In the multivariable linear mixed models (which used 98.7% of the study sample due to missing values), the L2C group had significantly decreased anxiety symptom scores from baseline to follow-up compared to the LIGHT control group [B (95% CI): −1.28 (−1.98, −0.58) vs. −0.37 (−0.93, 0.20), (interaction *P*= 0.019)]. Decreases in all other outcomes were greater in the L2C group as compared to the control, however, these differences were not statistically significant (Table 3).

The multivariable models also indicated several significant demographic variables when averaged across baseline and follow-up: males had significantly lower anxiety than females; having a baseline history of mental health difficulties was positively associated with depression, anxiety, and PSS symptom scores; and increased hurricane exposure and increased age were associated with increased PTSD symptoms scores.

## Discussion

The present analysis indicates that residents affected by Hurricane Sandy who presented with at least mild to moderate mental health burden and were linked into care exhibited a significant decrease in anxiety symptoms from baseline to follow-up. This decrease in anxiety persisted even after adjusting for factors known to modify the risk for mental health issues including time since the hurricane, demographic characteristics (e.g., race, ethnicity, gender), and prior mental health difficulties. There is evidence that differences in the decreases in anxiety symptom scores were greater in the L2C compared to the control group. For all other outcomes, although they were not significant, perhaps due to small sample sizes, the decreases in mental health symptom scores appear to be greater among those who were linked to care compared to those not linked. This suggests that linkage to mental health care in the wake of a natural disaster may be particularly effective in decreasing anxiety burden experienced by those exposed to such disasters. In combination, however, with the univariate findings, it is clear that providing post-disaster program support for struggling community members and facilitating linkage into mental health care, is integral in long-term disaster mental health response. It is not enough to just provide support during and in the immediate aftermath in the form of crisis counseling. Mental health interventions that are longer-lasting and that can be facilitated after the initial crisis has subsided are integral to positive community mental health.

The success of PR’s linkage to care model in reducing mental health symptoms, particularly anxiety, may have been due to several factors. First, PR was specifically targeted to those who were already displaying at least mild to moderate levels of symptoms of mental health difficulties increasing the likelihood that PR participants would be willing to link into care. Second, the screening and linkage process was individually tailored in a way that may have facilitated participant empowerment. As part of the screening process, participants could see for themselves that their mental health, including anxiety, symptomology was heightened. It is likely that seeing these scores and having the results of their baseline questionnaire discussed with them individually increased participants’ insight into their difficulties and perhaps empowered them to choose to address these difficulties.^39^ Once they agreed to participate in L2C, the coordinator reminded participants of paperwork to complete and of appointments to make, but it was the participants themselves that had to make and attend the initial appointment. This gave participants the opportunity and support they needed to take an active role in their mental health care as well as advocate on their own behalf. It is not clear, however, as to why only the change in anxiety symptoms was significantly different between the 2 groups and not the other mental health variables. This is most likely due to issues of statistical power resulting from low sample size. However, it is possible that anxiety was particularly heightened in this community after the hurricane and therefore particularly subject to treatment effects. This is consistent with another study conducted after Hurricane Sandy in the Rockaways in which anxiety was underscored as a major mental health issue as it was found to be experienced by 52% of the sample.^40^

Program sustainability is an important aspect of long-term mental health interventions if they are to be successful. In addition to increasing social worker capacity at a substance abuse and mental health clinic in Far Rockaway by providing financial support for additional staff, L2C provided local community mental health providers with potential clients for their clinics and practices. Mental health resources and provisions were limited in the Rockaways even before the hurricane.^41^ Scarcity of mental health providers only worsened after the hurricane as the mental health symptom burden increased as a result of the storm.^42^ L2C staff worked with local mental health care providers to understand what services their facilities were capable of providing and matched those providers with potential clients who were interested in linking into treatment services. L2C staff made sure that all provider information was up to date so that the participant would be able to directly link into mental health care while avoiding disconnected numbers and previous locations. Engagement by both the community and the providers indicate the potential utility in leveraging existing mental health treatment infrastructure in a community setting to address concerns among survivors. As other studies have also suggested, future research should examine the feasibility, acceptability, and cost-effectiveness of using existing mental health infrastructure as compared to building and implementing new interventions.^28^ An example of an existing structure that could be leveraged to address the mental health impact of natural disasters are workplace health promotion programs. Creating and supporting collaborations between occupational and public health has been a powerful tool during the COVID-19 pandemic and will most likely continue to be, as occupational health continues to move towards a more comprehensive approach of ‘total worker health’ recognizing the numerous factors, including psychosocial factors, that impact worker health, safety, and wellbeing.^43–45^

In addition, awareness around post-hurricane mental health issues increased in the community through L2C’s structure, which involved partnering with community groups to gain support for the program and enter locations to hold screenings. Increased mental health awareness in the community was also promoted by having PR staff speak at community events and by having PR’s mission and screening events publicized in the local newspaper and blogs.^46,47^ Furthermore, the program may have increased the sustainability of mental health service in that community as providers were able to see more clients and become more engaged in the locality. These results may help inform mental health intervention funding for future disasters and the allocation of resources to mental health treatment.

The results should be understood within the context of the study’s limitations. First, there were differences in follow-up time between the 2 surveys, although this was partially addressed in the models by using a heterogeneous autoregressive correlation structure to account for unequal time periods and adjust for the difference in months between baseline/follow-up surveys. Although PR participants had shorter follow-up time on average, their mental health symptom scores decreased at the same or at a greater rate than the LIGHT group. Although the 2 groups used the same eligibility criteria, baseline mental health scores were still higher for the L2C group. This could have resulted in greater regression to the mean for the L2C group, but diagnostics indicated that this was not the case. Another limitation is the potential selection bias from each cohort. Potential PR participants were aware that they may be linked to mental health care so those who wanted this service, possibly due to existing mental health symptoms, were more likely to be in the cohort. Additional selection bias occurred by those who elected to complete the follow-up surveys. Moreover, the current study was limited in that it utilized a pragmatic versus a randomized controlled trial (RCT) design. RCT designs are the gold-standard for evaluating the efficacy of interventions. Data from the current study supports the feasibility, acceptability, and initial effectiveness of the L2C intervention. As such, future work is needed to evaluate the efficacy of the L2C intervention during/after a disaster although ethically and pragmatically, it does not seem feasible to implement a post-disaster RCT. In addition, the actual psychotherapeutic treatment itself likely varied by participant and provider and was most likely the reason for any symptom improvements. However, due to confidentiality, study personnel were unable to collect any detailed treatment information. Finally, small sample sizes may have hindered the ability to detect significant interactions. Although significant decreases in mental health outcomes for PTSD, depression, and stress were seen in L2C and not in the control group, the interactions between the 2 groups on these variables were not significant. It may be that the study did not have enough power to statistically detect these effects due to small sample size.

Much can be learned from the overall approach to the implementation of Project Restoration. The study team’s collaborative relationship with the Rockaways community as well as the Project Restoration’s participant-specific strategy for linking into mental health care, facilitated the program’s success. This relationship allowed for adjustment of program implementation so that the methodology was most relevant and appropriate for use in the Rockaways community specifically. For example, the input of community members and stakeholders informed every aspect of the study process from recruitment strategy to language used when linking participants into care. Also, program staff were able to cultivate strong relationships with participants over the time it took to link participants into care. In turn, they were able to tailor resources to each participant to make sure their specific barriers to mental health care were addressed. Overall, Project Restoration demonstrates the strengths and challenges to implementing an intervention and testing its effectiveness in a real-world, post-disaster context when more rigorous methodological approaches such as an RCT are not feasible or ethical.

## Conclusion

In conclusion, linkage to care was associated with decreased symptoms of anxiety after Hurricane Sandy and may therefore help to decrease anxiety in affected communities. Findings from this study can inform the activities and policies of key stakeholders in the mental health disaster preparedness and mental health service provision communities. It is imperative that resources are in place to provide not just short-term, but longer-term mental health support to those impacted by a natural disaster. Further, it is necessary to understand both geographic and demographic vulnerabilities so that those most likely to experience negative mental health impacts of natural disasters are provided supportive services as quickly as possible.

## Supplementary Material

1

## Figures and Tables

**Table 1.** Comparison of baseline demographics between L2C and control group

Variable	Category	L2C^b^ (n = 52)^a^	Control (n = 63)^a^	*P* Value^c^

		*n* (%)	*n* (%)	
Participant Age (years)	mean (SD)^b^	48.21 (11.23)^d^	43.02 (19.42)^d^	0.138

Gender	Male	20 (38.46)	15 (23.81)	0.089

	Female	32 (61.54)	48 (76.19)	

Race	White	29 (55.77)	53 (84.13)	**0.001**

	Non-White	23 (44.23)	10 (15.87)	

Ethnicity	Non-Hispanic	38 (73.08)	48 (76.19)	0.702

	Hispanic	14 (26.92)	15 (23.81)	

Education	< HS^b^	17 (33.33)	3 (4.76)	**< 0.001**

	≥ HS^b^	34 (66.67)	60 (95.24)	

Prior MHD	No	18 (34.62)	45 (71.43)	**< 0.001**

	Yes	34 (65.38)	18 (28.57)	

Current MH^b^ Treatment	No	17 (32.69)	45 (84.91)	**< 0.001**

	Yes	35 (67.31)	8 (15.09)	

Hurricane Exposures Score		6.5 (4.0, 9.5)	5 (1.0, 10.0)	0.363

Time (months) between baseline survey and Hurricane Sandy	median (IQR^b^)	30.64 (25.72, 41.09)^d^	13.13 (12.60, 17.17)^d^	**< 0.001**

Time (months) between baseline and follow-up surveys	median (IQR^b^)	6.17(5.51, 8.26)^d^	16.91 (15.77, 22.80)^d^	**< 0.001**

Baseline Problem Drug Use	No	24 (46.15)	40 (76.92)	**0.001**

	Yes	28 (53.85)	12 (23.08)	

Baseline Current Smoker	No	24 (48.00)	53 (84.13)	**< 0.001**

	Yes	26 (52.00)	10 (15.87)	

Baseline Problem Alcohol Use	No	29 (55.77)	33 (52.38)	0.717

	Yes	23 (44.23)	30 (47.62)	

Baseline PTSD symptom score	mean (SD^b^)	42.50 (15.68)^d^	30.91 (15.80)^d^	**< 0.001**

Baseline Depression symptom score	mean (SD^b^)	3.02 (2.20)^d^	2.05 (1.98)^d^	**0.017**

Baseline Anxiety symptom score	mean (SD^b^)	3.71 (2.23)^d^	2.60 (2.08)^d^	**0.010**

Baseline Perceived stress symptom score	mean (SD^b^)	22.96 (7.53)^d^	19.46 (7.46)^d^	**0.013**

a Numbers may not add to total due to missing values in each category

b L2C = Link to Care; MH = mental health; HS = high school; SD = standard deviation; IQR = interquartile range

c *P* value from Chi-square tests for categorical variables and Mann-Whitney rank sum test for age, time between baseline survey and Hurricane Sandy, hurricane exposure score, and mental health symptom scores

d Mean, SD or Median, IQR presented instead of n, %

**Table 2.** Distribution of outcomes between L2C and control group

Variable	L2C^b^ (N = 52)^a^			Control (N = 63)^a^		
	Baseline	Follow-up	*P* Value^c^	Baseline	Follow-up	*P* Value^c^

	*n* (%)	*n* (%)		*n* (%)	*n* (%)	
Problem Drug Use	28 (53.85)	18 (39.13)	**0.046**	12 (23.08)	11 (17.74)	0.739

Current Smoker	26 (52)	25 (51.02)	0.480	10 (15.87)	9 (14.52)	0.655

Problem Alcohol Use	23 (44.23)	17 (37.78)	0.366	30 (47.62)	37 (58.73)	0.071

PTSD symptom score, mean (SD)	42.50 (15.68)	35.52 (14.31)	**0.008**	30.91 (15.80)	27.78 (13.71)	**0.014**

Depression symptom score, mean (SD)	3.02 (2.20)	2.35 (1.95)	**0.040**	2.05 (1.98)	1.67 (1.85)	0.131

Anxiety symptom score, mean (SD)	3.71 (2.23)	2.5 (2.12)	**< 0.001**	2.60 (2.08)	2.27 (1.83)	0.133

Perceived stress symptom score, mean (SD)	22.96 (7.53)	19.25 (7.82)	**< 0.001**	19.46 (7.46)	17.29 (8.86)	**0.014**

a Numbers may not add to total due to missing values in each category

b L2C = Link to Care; PTSD = Post-traumatic Stress Disorder; SD = Standard Deviation

c *P* value from McNemar’s test for categorical variables and Wilcoxon Sign Rank test for mental health measures

**Table 3.** Adjusted parameter estimates of covariates associated with mental health symptom scores - linear mixed models

Effect	PTSD symptom score	Depression symptom score	Anxiety symptom score	Perceived stress score
	Adj. B (95% CI)	Adj. B (95% CI)	Adj. B (95% CI)	Adj. B (95% CI)
Intercept	18.00 (2.35, 33.66)	0.60 (−1.51, 2.71)	1.64 (−0.40, 3.69)	11.96 (3.21, 20.71)

L2C	8.53 (−1.79, 18.84)	0.66 (−0.77, 2.09)	1.38 (0.00, 2.76)	1.99 (−3.77, 7.75)

Period	−3.13 (−6.56, 0.3)	−0.28 (−0.75, 0.19)	−0.37 (−0.85, 0.11)	−2.17 (−4.03, −0.32)

L2C*Period^a^	−3.99 (−9.12, 1.14)	−0.48 (−1.24, 0.27)	**−0.91 (−1.68, −0.15)**	−1.57 (−4.35, 1.21)

Age (years)	0.13 (0.00, 0.26)	0.01 (−0.01, 0.02)	0.01 (−0.01, 0.02)	0.04 (−0.03, 0.11)

Gender (Male vs. Female)	−2.55 (−7.05, 1.96)	−0.33 (−0.92, 0.27)	**−1 (−1.57, −0.43)**	−2.52 (−5.03, 0.00)

Race (Other vs. White)	−1.57 (−7.18, 4.04)	−0.07 (−0.84, 0.7)	−0.62 (−1.38, 0.15)	−1.04 (−4.17, 2.1)

Ethnicity (Hispanic vs. Non-Hispanic)	2.73 (−2.67, 8.13)	0.69 (−0.06, 1.43)	0.50 (−0.22, 1.21)	1.88 (−1.14, 4.89)

Education (Greater than HS vs. HS or less)	−4.21 (−10.35, 1.94)	−0.87 (−1.73, 0.00)	−0.38 (−1.22, 0.47)	−2.4 (−5.84, 1.03)

Prior MH History (Yes vs. No)	**4.79 (0.22, 9.37)**	**0.86 (0.23, 1.48)**	**1.12 (0.51, 1.73)**	**3.69 (1.14, 6.25)**

Hurricane Exposure Score	**1.14 (0.63, 1.64)**	0.03 (−0.03, 0.10)	0.03 (−0.03, 0.09)	0.19 (−0.09, 0.47)

Time (months) between baseline survey and Hurricane Sandy	0.09 (−0.28, 0.45)	0.03 (−0.02, 0.08)	0.00 (−0.05, 0.05)	0.15 (−0.05, 0.36)

Time (months) between baseline and follow-up surveys	0.11 (−0.36, 0.58)	0.05 (−0.02, 0.11)	0.04 (−0.02, 0.1)	0.21 (−0.06, 0.47)

*Note*. L2C = Link to Care; PTSD = Post-traumatic Stress Disorder; HS = high school; MH = mental health; adj.B = adjusted parameter estimate; SE= standard error

a Interaction between L2C and period
