# Supplementary material for: Assessing the Feasibility and Effectiveness of a Linkage Into Mental Health Care Program for Adults Affected by Hurricane Sandy
Source: Disaster Med Public Health Prep. Author manuscript; Available in PMC 2023 Mar 5. (PMC9905213; doi:10.1017/dmp.2022.176)
Supplement: 1 [file NIHMS1822942-supplement-1.docx]

**S1 Table. Screening criteria to determine eligibility for Project Restoration**

| **Mental Health Outcomes** | **Measure Used** | **Description** | **Screen-in Criteria** |
| --- | --- | --- | --- |
| Problem Alcohol Use | NIAAA* guidelines on binge drinking |  | Usage response of **“Monthly”** or more frequently |
|  |  |  |  |
| Recreational Drug Use | NIDA* guidelines |  | Usage response of **“Monthly”** or more frequently |
|  |  |  |  |
| Family Substance Abuse |  |  | Answer of “**Yes”** to Are you currently being impacted by the substance use of a family member? |
|  |  |  |  |
| Perceived Stress | Perceived Stress Scale | 10 items (range 0-4) | Minimum of FIVE items with a **3** or **greater** response |
|  |  |  |  |
| Depression | Patient Health Questionnaire-4 (items 1-2) | 2 items (range 1-4) | Average score of **2 or above** |
| Anxiety | Patient Health Questionnaire-4 (items (3-4) | 2 items (range 1-4) | Average score of **2 or above** |
| Posttraumatic Stress Disorder | Posttraumatic Stress Disorder Checklist- Specific | 17-items (range 1-5) | Minimum of EIGHT items with a 3 or greater response or |
|  |  |  | Minimum or FOUR items with a 5 response |

*NIAAA = National Institute on Alcohol Abuse and Alcoholism NIDA = National Institute on Drug Abuse

**S2 Table. Comparison of demographic and mental health characteristics of participants who completed follow-up questionnaires to LIGHT and PR study samples.**

|  |  | **LIGHT** | | | | **PR** | | | |  |
| --- | --- | --- | --- | --- | --- | --- | --- | --- | --- | --- |
| **Variable** | **Category** | **Total (N=658)** | **Control (n=128)^a^** | **Control (n=63)^a^** | **Total (N=1011)** | | **Screened In (n=488)^a^** | **Participated (n=143)^a^** | **L2C (n=52)^a^** | |
|  |  | **n (%)** | **n (%)** | ***n (%)*** | **n (%)** | | **n (%)** | **n (%)** | **n (%)** | |
| Age (years), Mean, SD^b^ |  | 47.82 (23.42) | 49.59 (20.95) | 43.02 (19.42) | 45.18 (15.81) | | 44.84 (13.80) | 45.43 (12.71) | 48.21 (11.23) | |
| Gender | Male | 234 (35.62) | 28 (21.88) | 15 (23.81) | 439 (43.47) | | 224 (45.90) | 62 (43.36) | 20 (38.46) | |
|  | Female | 423 (64.38) | 100 (78.13) | 48 (76.19) | 571 (56.53) | | 264 (54.10) | 81 (56.64) | 32 (61.54) | |
| Race | White | 462 (70.86) | 93 (73.81) | 53 (84.13) | 390 (39.39) | | 187 (39.12) | 66 (53.22) | 29 (55.77) | |
|  | Non-White | 190 (29.14) | 33 (26.19) | 10 (15.87) | 600 (60.61) | | 291 (60.88) | 74 (46.78) | 23 (44.23) | |
| Ethnicity | Non-Hispanic | 517 (79.29) | 104 (82.54) | 48 (76.19) | 819 (82.73) | | 393 (82.22) | 108 (44.26) | 38 (73.08) | |
|  | Hispanic | 135 (20.71) | 22 (17.46) | 15 (23.81) | 171 (7.27) | | 85 (17.78) | 33 (51.56) | 14 (26.92) | |
| Education | < HS | 42 (6.56) | 7 (5.51) | 3 (4.76) | 212 (21.77) | | 119 (25.27) | 46 (54.12) | 17 (33.33) | |
|  | > HS | 598 (93.44) | 120 (94.49) | 60 (95.24) | 762 (78.23) | | 352 (74.73) | 94 (43.12) | 34 (66.67) | |
| Prior MH History | No | 508 (77.44) | 100 (78.13) | 45 (71.43) | 707 (70.21) | | 280 (57.85) | 50 (35.21) | 18 (34.62) | |
|  | Yes | 148 (22.56) | 28 (21.88) | 18 (28.57) | 300 (29.79) | | 204 (42.15) | 92 (55.09) | 34 (65.38) | |
| Current MH Treatment | No | 487 (89.85) | 83 (90.22) | 45 (84.91) | 789 (79.94) | | 320 (68.23) | 50 (29.24) | 17 (32.69) | |
|  | Yes | 55 (10.15) | 9 (9.78) | 8 (15.09) | 198 (20.06) | | 149 (31.77) | 91 (69.47) | 35 (67.31) | |
| Hurricane exposure score, | Median, IQR^b^ | 3 (1 - 6) | 3.5 (1-8) | 5 (1, 10) | 5 (2-9) | | 6 (3 – 10) | 6 (3-9) | 6.5 (4-9.5) | |
| Time (months) between baseline survey and Hurricane Sandy, Median, IQR^b^ |  | 13.68 (12.60-17.96) | 13.13 (12.30-16.71) | 13.13 (12.60, 17.17) | 31.84 (23.55-41.09) | | 31.84 (22.86 – 41.48) | 31.84 (24.67-40.86) | 30.64 (25.70-41.09) | |

*Note.* L2C= Link to Care, MH= mental health, HS= high school, SD= standard deviation, IQR= interquartile range

^a^ Numbers may not add to total due to missing values in each characteristic.

^b^ Mean, SD or Median, IQR presented instead of n, %
